# Perceived Risk of Fish Consumption in a Low Fish Consumption Country

**DOI:** 10.3390/foods9091284

**Published:** 2020-09-12

**Authors:** Ágoston Temesi, Dawn Birch, Brigitta Plasek, Burak Atilla Eren, Zoltán Lakner

**Affiliations:** 1 Department of Food Chain Management, Institute of Agribusiness, Szent István University, 1118 Budapest, Hungary; plasek.brigitta@etk.szie.hu (B.P.); eren.burak.atilla@hallgato.uni-szie.hu (B.A.E.); lakner.zoltan@etk.szie.hu (Z.L.); 2 USC Business School, University of the Sunshine Coast, Sippy Downs, Queensland 4556, Australia; dbirch@usc.edu.au

**Keywords:** perceived risk, functional risk, psychological risk, social risk, physical risk, negative past experiences, structural equation modeling, consumer behavior

## Abstract

Among the numerous health benefits of fish consumption, perhaps the most recognized is the role of omega-3 fatty acids in the prevention of cardiovascular disease. Cardiovascular disease is prevalent in Hungary, which has the lowest fish consumption in Europe. Increasing fish consumption is the aim of most European countries and given the high incidence of cardiovascular disease in Hungary, it is of particular importance. A significant reduction of the VAT for fish in 1 January 2018 aimed to increase fish consumption in Hungary. However, despite reduced VAT, the price of fish in Hungary rose from 2017 to 2018. The aim of our research is to explore perceived risks that serve to exacerbate Hungarian consumers’ low fish consumption, and to measure their effects to identify potential strategies to most effectively increase fish consumption. We applied partial least squares structural equation modeling (PLS-SEM) to analyze responses provided by 1042 survey participants (collected with face-to-face interviews, using quota sampling in 2014) to explore variables of fish consumption associated with perceived risk including psychological, physical, social, and functional risks. Our model is the first one that applies detailed perceived risk categories to measure those effects on low fish consumption. The results indicate that psychological risk associated with negative past experiences have both a direct, and through functional risk, an indirect significant negative effect on fish consumption. Conversely, neither social nor physical risk impede Hungarian fish consumption. We conclude that the seafood industry could benefit from targeted interventions that seek to reduce functional risk-perception of the person responsible for preparing fish in the household.

## 1. Introduction

Whilst in 2004, Olsen noted a surprisingly low volume of research on fish consumption from the perspective of marketing and consumer behavior [1], a more recent review by Carlucci et al. (2015) identified 49 relevant studies [2]. In more recent times, consumer research on barriers to fish consumption have been conducted across the globe [3] and in specific continents including Europe [4,5], Africa [6], Asia [7], Australia [8], South-America [9,10]. In their review, Carlucci et al. (2015) identified the main barriers of fish consumption to be associated with unpleasant sensory qualities of fish, low convenience, consumer lack of confidence in selecting and preparing fish, concerns about potential health risks, low availability of fish and high prices [2].

Understanding barriers to fish consumption is especially important in countries where consumption is markedly low, and in some cases, decreasing. Of concern, Eumofa (2019) data reveals long-range stagnation of fish consumption in some Central-European countries. For example, Hungary has the lowest annual per capita fish consumption in Europe (5.6 kg) with only 5% of expenditure on animal proteins in 2018 being for fish and seafood. Likewise, only 9% of expenditure on animal proteins in the Czech Republic in 2018 was spent on fish and seafood [11].

Fish consumption is important due to numerous positive health benefits for example, reduced incidence of disease such as breast cancer (e.g., [12]), sarcopenia [13], and mental illnesses such as depression [14], as well as, neurological diseases (e.g., [15]). In particular, researchers emphasize the preventive role of omega-3 fatty acids and role of fish consumption in reducing cardiovascular disease [16,17,18,19]. We argue there may be a direct correlation between health problems in Hungary and very low fish consumption, with Hungary reporting the worst data for cardiovascular disease within the European Union [20,21].

**Figure 1:**
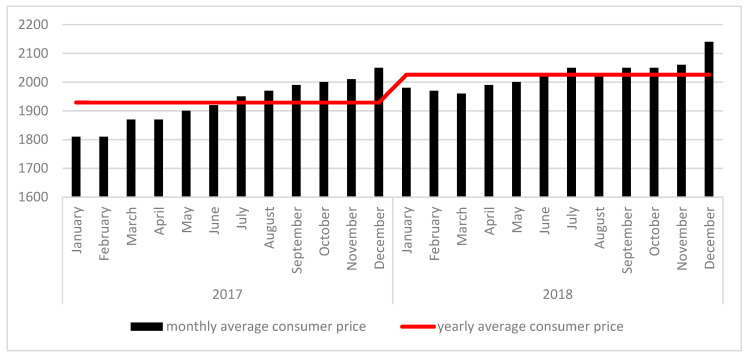
Monthly and yearly national average consumer price of a carp slice or fillet HUF/kg Source: [24].

Seeking to increase fish consumption, the Hungarian government reduced the VAT for fish by 22% in 1 January 2018. In Hungary, carp is one of the most popular species of fish [22,23]. Changes in the price of a slice of carp, in monthly intervals in 2017 and 2018 (the year before and after the reduced VAT) are illustrated in Figure 1.

The data reveals that the reduction of VAT in January 2018 successfully lowered the rising price of a carp slice; however by July 2018, only 7 months later, the price reached the previous 2017 price level. Hence, the reduction of VAT only temporarily reduced and then constrained the price, potentially explained by opportunistic behavior on behalf of Hungarian fish mongers. Given the latest fish consumption data available from FAO, Eumofa, and EC are from 2017, it is not possible to state whether the reduced VAT led to any increase in fish consumption in Hungary. However, while price (representing financial risk for the consumer), has been identified as a major barrier to fish consumption, other risks including functional, social, physical and psychological risks associated with fish consumption also influence fish consumption [25,26], and these risks are the focus of this paper.

### 1.1. Conceptual Model

Numerous studies have investigated reasons for lower-than-optimal fish consumption across the globe [2]. Several studies have explored the effect of food neophobia on fish consumption, and concluded that a higher degree of food neophobia negatively influenced fish consumption both for children [27,28] and adults [28,29,30,31,32]. Jaeger et al. [32] showed in the case of 112 analyzed foods—among them different forms of fish—that food neophobia has a significant effect on both the frequency of the given food’s intake and on the preferences related to it. Many papers have been premised on the theory of planned behavior [33], for example, in Indonesia [34], Peru [10], Croatia [35], Bangladesh [36], Vietnam [37,38] and Belgium [39]. Data analysis for fish consumption studies has frequently involved the use of structural equation modeling (SEM) [38,40,41,42,43,44,45]. SEM was used by Pieniak et al. [46] and Siddique [36] and Schaefer et al. [47] used regression based on the variables of “risk perception” and “perceived risk”, however, these two variables only explained a fraction of the effect. In an Australian study, Birch and Lawley [25] applied perceived risk theory [48,49] to explore the role of various categories of perceived risk on fish consumption, but they did not attempt modeling. Categories of perceived risk were first identified by Jacoby and Kaplan [50], distinguishing functional (or performance risk), physical, social, financial and psychological risks. In a consumer context, Murphy and Enis (p. 31 [51]) define risk as the “monetary and nonmonetary price of the product” with financial risk being monetary and social, psychological, physical, functional being nonmonetary.

In line with a study of perceived barriers to fish consumption in Australia conducted by Birch and Lawley [25], we investigate non-monetary risks associated with fish consumption in Hungary including physical, social, psychological (negative past experiences) and functional (occurring during cooking) risks.

#### 1.1.1. Physical Risks

Fish consumption may involve various physical risks including choking on bones, allergic reactions, spoiled fish and contaminants such as heavy metals [25,47,52]. An increase in consumers’ perception of the physical risk of consuming fish may be expected as freshwater contamination becomes better detected [53,54,55,56]. While communication of potential physical risk associated with fish consumption (e.g., mercury) is important [57], Anual et al. argue that consumers should be informed about contaminants in a way that equips them with the knowledge to more effectively manage the risk rather than resulting in decreased fish consumption [58]. Given potential perceived physical risks associated with fish consumption, we hypothesize:

**Hypothesize 1 (H1).** **** Physical risks directly and negatively influence fish consumption.

#### 1.1.2. Social Risks

Fish consumption studies have investigated the role of social norms and social risks in mitigating fish consumption [5,25,39,59,60,61]. For example, in a Belgian study (*n* = 429) conducted by Verbeke and Vackier [39], one quarter of respondents not living alone indicated that they served a fish dish less frequently because of the resistance of the household members, and specifically concluded that the presence of a teenager in the household negatively influences fish consumption. Likewise, in an Australian study (*n* = 899), Birch and Lawley [25] found that nearly a third of the Australian respondents not living alone also served fish less frequently if other members of the household disliked fish. In Pinho et al.’s research [5], the barrier of the “taste preference of family and friends” proved to be a significant factor with households of three or more people. Zhou et al. [61] confirmed the findings of Myrland et al. [59], and Verbeke and Vackier [39] and concluded that the presence of a teenager in the household negatively influences fish consumption. Given the potential for dislike of fish by others to reduce fish consumption, we hypothesize:

**Hypothesize 2 (H2).** **** Social risks directly and negatively influence fish consumption.

#### 1.1.3. Psychological Risks

Past experience influences intention to eat fish [42]. Fish consumption in childhood has been found to influence fish consumption in adulthood with studies indicating that regular childhood fish consumption leads to higher fish consumption as an adult (e.g., [41,62,63]). Conversely, too frequent fish consumption in childhood may result in aversion towards fish as a food in adulthood [62]. Negative past experiences associated with dislike of the sensory qualities of fish (bones, smell, appearance, taste, texture, etc.) have been found to lead to lower fish consumption [2,8,25,59]. In Hungary, a key source of unpleasant sensorial experiences may potentially be linked to consumption of primarily freshwater fish (that may have a muddy taste) [6], given Hungary is landlocked country. Given negative past experiences may influence fish consumption, we hypothesize:

**Hypothesize 3 (H3).** **** Psychological risk associated with negative past experiences directly and negatively influences fish consumption.

#### 1.1.4. Functional Risks

Functional risk has been found to be a major barrier to fish consumption [25]. Numerous studies highlight the importance of knowledge in increasing fish consumption [1,4,43,64]. Hence, increasing consumer knowledge and confidence around selecting, cooking and serving fish (especially younger consumers) may lead to increased fish consumption [8,63]. Lack of knowledge and confidence may arise from low familiarity that has been found, for example, to be a key barrier to consumption of seafood such as shrimp and mussels [65]. Contini et al. emphasized the role of cooking skills on intention to consume fish [40], however, lack of knowledge of the person responsible for cooking in terms of fish preparation may not have the same significance in all countries, and rather may be more closely related to consumption frequency [66]. In our study, we specifically explore the functional risks that emerge during the preparation of fish dishes by the person responsible for cooking within the household. Given the potential for functional risk to influence fish consumption, we hypothesize:

**Hypothesize 4 (H4).** **** Functional risk arising during the preparation of fish directly and negatively influences fish consumption.

#### 1.1.5. Interaction Effects

Badr. et al.’s study revealed that consumers regard the preparation of freshwater fish to be particularly difficult, requiring knowledge and skills [6]. The lack of these can easily result in improperly prepared fish meals. Poorly prepared fish arising from functional risk likely leads to a less than pleasing consumption experience, thus increasing the likelihood of psychological risk. According to the results of Laureati et al. [28] whether kids like a fish dish depends greatly on its cooking method. They connect all of this to neophobia and note that choosing the right recipe can significantly contribute to reducing it. Hence, we hypothesize:

**Hypothesize 5 (H5).** **** Functional risk that arises during cooking directly and positively influence psychological risk due to negative past experiences.

Poorly prepared fish may also lead to lower familiarity and acceptance of fish by other members of the household thus increasing social risk due to food acculturation effects. The effect of which is amplified further as the acceptance of unknown fish and fish meals will be more difficult due to higher perceived risk [67] and may result in the younger generation regarding the preparation of fish meals as even more difficult [39,45,68]. Hence, we hypothesize:

**Hypothesize 6 (H6).** **** Functional risk that arise during preparation directly and positively influence (facilitate) the development of social risk.

**Figure 2:**
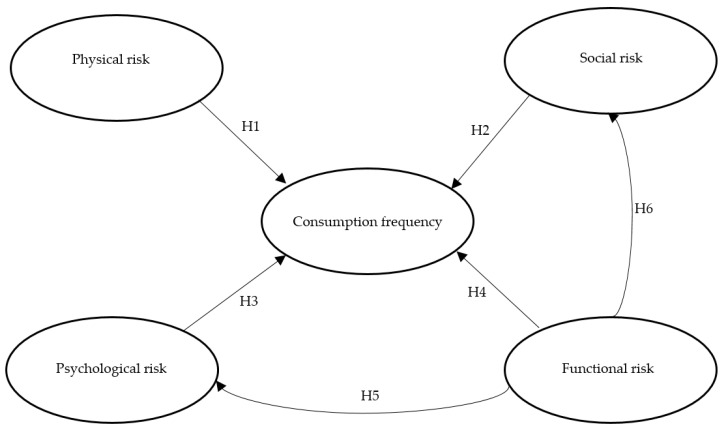
Conceptual framework.

Figure 2 presents the conceptual model for the study indicating direct and indirect effects.

The aim of the present research is to apply the theory of perceived risk and contribute to the increasingly sophisticated modelling that seeks to explain complex fish consumption behavior. The context for the study is in a very low fish consumption country, namely Hungary, with the aim of identifying the role of perceived risk and inform strategies for reducing identified perceived risks in order to increase fish consumption.

## 2. Materials and Methods

A face to face state-wide paper-based survey of 1063 Hungarian consumers was conducted between 22nd September to 10th October 2014 in public places of 8 big cities of Hungary, namely in Budapest, Székesfehérvár, Pécs, Győr, Miskolc, Debrecen, Szolnok, and Szeged, using a standardized questionnaire. Due to the refusal to answer certain questions, we discarded the responses of 21 respondents, and thus analyzed the data from 1042 respondents. The respondents were motivated with a small non-food gift for participating in the survey. Before completing the survey, the respondents provided verbal consent to their answers being recorded. At the beginning of the survey we also informed them in writing that their answers will later be analyzed, but at the same time the responses would remain anonymous, and we did not collect any specific demographic data about the respondents. They had the option to refuse to answer any question or stop answering the survey at any point. The data is representative of the general population with respect to age and gender as a result of quota-sampling. A respondent profile is presented in Table 1.

In the two main parts of the questionnaire, we queried about the frequency of fish consumption and agreement with attitude statements.

Attitude statements for the survey were replicated or modified to the Hungarian consumption context based on the work of Birch and Lawley [25]. The degree of agreement was measured on a 5-point Likert-scale with ends labelled 1 = completely disagree and 5 = completely agree. The research focused on barriers of fish consumption and all the attitude statements are associated with perceived risk. The following attitude statements were included in the investigation (Table 2):

Partial least square based structural equation modeling (PLS-SEM) was performed with the help of the SmartPLS software [70]. We built a reflective model, with frequency of consumption as a dependent variable measured with the question—How often did you consume a whole portion (10–15 dekagrams) of fish in the past year?

## 3. Results

### 3.1. Frequency of Fish Consumption

Typically, health guidelines recommend consumption of two servings of fish per week. For the grouping of frequency of consumption, we used the categorization of Birch and Lawley [25], regular fish consumers (2–3 times per week to at least once a week), light fish consumers (about once per fortnight), and very light fish consumers (once per month). Confirming the very low average fish consumption in Hungary, 12% (*n* = 120) of respondents report never consuming fish. Only 47% of respondents consume fish at least once per month. We categorized respondents who consume fish less than once per month but at least once a year (*n* = 440, 42%) as “extremely light” fish consumers. They may be the ones who typically, but not exclusively, consume the traditional Christmas fish dishes (fish soup, carp in breadcrumbs) in Hungary, so they are familiar with fish dishes, but consume them only on holidays. Very light fish consumers (*n* = 263) accounted for 25% of those surveyed and light fish consumers accounted for a further 10% (*n* = 107), while regular fish consumers (*n* = 112) only accounted for 11% of the respondents. Consumption frequency characteristics of the sample are introduced in Table 3.

### 3.2. Measurement of Model

The attitude statements of the research tested were built into the model. The factor loadings of the items and the values belonging to the background variables of the model are shown in Table 4.

The values of the model confirm its reliability. Composite Reliability values fall between 0.767 and 0.823 and exceed the expected value of 0.7 in all cases [71]. The values of average variance extracted (AVE) vary between 0.505 and 0.565, and thus exceed the expected score of 0.5 [72]. Finally, Cronbach’s alpha scores exceed 0.7 [73]. Although some factor loadings do not exceed 0.7, retaining them is in accordance with the recommendation of Hair et al. [74], who argue that items with factor loadings between 0.4 and 0.7 should be examined to determine whether discarding them will result in worse indices for the model. Moreover, in all cases, the values substantially exceed the value (<0.5) that Bagozzi and Yi consider the threshold for rejection [75]. The Collinearity Statstics show, that the VIF values for all items are below the threshold of 3 [72].

Considering the fact that we built a reflective model, the indices of the structural model were calculated with Consistent PLS Algorithm. Our results indicate that the model is a good fit (SRMR = 0.061, NFI = 0.856), and is consistent with the recommendation of Hu and Bentler [76], in that the SRMR score should remain below 0.8. Table 5 and Table 6 show the results of the discriminant validity. Table 5 displays the Fornell-Larcker test of discriminant validity, while Table 6 shows the Heterotrait-Monotrait Ratio (HTMT).

Table 5 shows that the specific values of the square roots of the average variance extracted in the constructs are in all cases higher than the correlation values in the same columns or rows [77], whereas the values in Table 6 are remarkably lower than the 0.85 threshold [78], which means that discriminant validity has been established.

### 3.3. Structural Model Assessment

**Figure 3:**
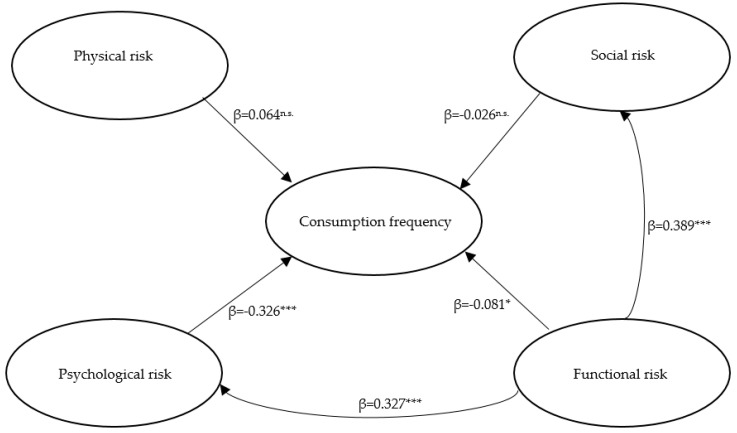
Results of structural equation modeling. β = standardized regression coefficient; * *p* < 0.05, *** *p* < 0.001, ^n.s.^ not significant.

Bootstrapping procedure has been used to test level of significance and t statistics. Our first hypothesis (H1), the direct and negative relationship between the perception of physical risk and consumption frequency was not confirmed by our model (β = 0.064, *p* = 0.070). Similarly, the hypothesized direct and negative relationship between the perception of social risk and consumption frequency (H2) could also not be verified (β = −0.026, *p* = 0.465). The direct and negative effects of unpleasant experiences as psychological risk on consumption frequency (H3) were verified (β = −0.326, *p* < 0.001), as was the perceived functional risk during cooking (H4) (β = −0.081, *p* = 0.043), although Cohen’s f-square is very low. The hypothesized relationships between risks were also confirmed in our research. Our results show a direct and positive relationship between functional risk perceived during cooking and the psychological risk associated with negative past experiences (H5) (β = 0.327, *p* < 0.001), as well as between functional risk perceived during cooking and the perception of social risk (H6) (β = 0.389, *p* < 0.001). Table 7 and Figure 3 summarize our results.

The values of R^2^, adjusted R^2^ and Q^2^ in Table 8 illustrate the explanatory power of the model.

The explanatory power of the model for consumption frequency is 13% (adjusted R^2^ = 0.130), which is low but acceptable see [71,79]. Stone-Geisser’s Q^2^ values [74,80,81] are greater than 0, which means that each element of the endogenous constructs has predictive relevance. (Psychological risk = 0.043, Consumption frequency = 0.102, Social risk = 0.064).

## 4. Discussion

The aim of this research was to explore perceived risks that hinder fish consumption and their relative importance in Hungary, which has the lowest fish consumption in Europe. Our model is the first one that applies detailed perceived risk categories to measure those effects on low fish consumption. The structural equation model built to test the hypotheses did not support a direct and negative effect of either physical or social risk on fish consumption. Interestingly and contrary to expectations, perceived physical risk (perceived lack of hygiene, contamination, or spoiled fish) has a slight positive relationship with fish consumption. This may be explained by higher levels of involvement with fish and greater knowledge about fish among people who consume more fish. However, this awareness of potential physical risk is managed and does not hinder their fish consumption.

We observed a direct and negative relationship between functional risk arising during preparing fish and psychological risk associated with negative past experiences and frequency of consumption. Based on our model, we did not find a direct effect of functional risk on frequency of consumption, as Cohen’s f² value is extremely low. However, indirectly, through negative past experiences, functional risk has a significant negative effect on frequency of consumption. This is consistent with the findings of Laureati et al. [28], who find cooking methods and choosing the right recipe to be of key importance regarding young people’s acceptance of fish dishes.

Likewise, while functional risk increases perceptions of social risk, functional risk does not have an indirect effect on the frequency of fish consumption through social risk. Thus it appears that in effect, fish consumption in Hungary is not influenced by the fact whether others in the household like fish dishes.

Our results indicate that the fish industry could benefit from targeted interventions that reduce perceptions of functional risk. This would involve focusing on educating the person responsible for preparing fish within households to improve their skills in preparing fish dishes, or semi-prepared products, which can help boost their confidence and be more successful. This will likely lead to higher levels of household consumption of fish leading to greater familiarity and acceptance of fish for meals, thus mitigating social risk. Moreover, increased self-efficacy with fish preparation will likely lead to better (sensory) experiences and thus reduce perceived psychological risk associated with fish.

## 5. Limitations and Further Research

Our study is the first to use perceived risks as background variables for structural equation modeling to explain fish consumption frequency. The explanatory power of the model (R^2^) is small, as we used the elements of perceived risk that only partially explain fish consumption and willingness to purchase fish. Our findings are consistent with Siddique [36] who found the effect of perceived risk on dry fish consumption to be 18%. In accordance with the aim of the research, further research will build broader models including other constructs explaining fish consuming behavior, such as habit, or food neophobia. Further researches will focus not only on risks but other barriers and also drivers of fish consumption will show better explanatory power when using new factors. This study focused on Hungary based on concern about being the lowest fish consumption country within Europe and high incidence of cardiovascular disease that would benefit from increased fish consumption. Future studies could test the model in other countries with similarly low fish consumption and health issues.

## Figures and Tables

**Table 1:** Demographic and income properties of respondents (*n* = 1042).

Variables		Composition of the Sample	Composition of the Population *
Gender	Male	46.8%	46.9%
	Female	53.0%	53.1%
	Missing	0.2%	
Age group	18–25	11.6%	11.5%
	26–35	17.8%	15.3%
	36–45	18.1%	19.5%
	46–55	16.4%	15.8%
	56 or older	35.9%	37.9%
	Missing	0.2%	
Education	Elementary	6.6%	
	Trade/vocational	16.3%	
	Secondary	32.1%	
	Tertiary	42.6%	
	Missing	2.3%	
Region	Northern Hungary	6.9%	11.6%
	Northern Great Plains	12.8%	14.9%
	Southern Great Plains	14.7%	12.8%
	Central Hungary	32.2%	30.7%
	Central Transdanubia	10.0%	10.8%
	Western Transdanubia	10.7%	10.0%
	Southern Transdanubia	8.5%	9.1%
	Missing	4.2%	
Perceived income status	Very tight	2.5%	
	Tight	11.7%	
	Average	58.4%	
	Good	20.6%	
	Very good	4.0%	
	Missing	2.7%	

* Source: [69].

**Table 2:** Items and constructs.

Psychological risk
I like the taste of fish—reverse coded
I came to like fish already as a child–reverse coded
I have had good experiences in eating sea fish in the past – reverse coded
I have had good experiences in eating freshwater fish in the past–reverse coded
Physical risk
I am concerned that spoiled fish will be sold to me
I am concerned that fish may not have been handled in a hygienic way
I am concerned that fish contains a lot of contaminants from sea
I am concerned that fish contains a lot of contaminants from freshwaters
Functional risk
The person who cooks in our household does not know how to prepare freshwater fish
The person who cooks in our household does not know how to prepare saltwater fish
It is hard for the person who cooks in our household to bring him/herself to cook from fish that (s)he does not know
Social risk
Other adults in my household do not like fish
One or more children in my household do not like fish
Resistance by other members of my household makes it hard to serve fish as often as I want

**Table 3:** Consumption frequency of the sample (*n* = 1042).

Consumption Frequency		*n*	%
Regular	(2–3 times per week to at least once a week)	112	11%
Light	(About once per fortnight)	107	10%
Very light	(Once per month)	263	25%
Extremely light	(Less than once per month but at least once a year)	440	42%
Never	(Never)	120	12%

**Table 4:** Construct reliability and validity.

Construct and Indicators	Factor Loading
Psychological risk (CR = 0.823, AVE = 0.539, CA = 0.823)	
I like the taste of fish—reverse coded	0.776
I came to like fish already as a child—reverse coded	0.634
I have had good experiences in eating sea fish in the past—reverse coded	0.733
I have had good experiences in eating freshwater fish in the past—reverse coded	0.783
Physical risk (CR = 0.803, AVE = 0.505, CA = 0.801)	
I am concerned that spoiled fish will be sold to me	0.667
I am concerned that fish may not have been handled in a hygienic way	0.730
I am concerned that fish contains a lot of contaminants from sea	0.670
I am concerned that fish contains a lot of contaminants from freshwaters	0.772
Functional risk (CR = 0.767, AVE = 0.525, CA = 0.762)	
The person who cooks in our household does not know how to prepare freshwater fish	0.782
The person who cooks in our household does not know how to prepare saltwater fish	0.729
It is hard for the person who cooks in our household to bring him/herself to cook from fish that (s)he does not know	0.657
Social risk (CR = 0.789, AVE = 0.565, CA = 0.787)	
Other adults in my household do not like fish	0.582
One or more children in my household do not like fish	0.697
Resistance by other members of my household makes it hard to serve fish as often as I want	0.933

* CR = composite reliability, AVE = average variance extracted, CA = Cronbach’s alpha.

**Table 5:** Fornell-Larcker test of discriminant validity.

	Functional Risk	Psychological Risk	Consumption Frequency	Physical Risk	Social Risk
Functional risk	**0.724**				
Psychological risk	0.327	**0.734**			
Consumption frequency	−0.179	−0.353	**1.000**		
Physical risk	0.284	0.127	−0.005	**0.711**	
Social risk	0.389	0.349	−0.157	0.214	**0.751**

Square roots of the average variance extracted (AVE) shown on diagonal (in bold).

**Table 6:** Heterotrait-monotrait (HTMT) criterion for discriminant validity.

	Functional Risk	Psychological Risk	Consumption Frequency	Physical Risk	Social Risk
Functional risk					
Psychological risk	0.330				
Consumption frequency	0.179	0.352			
Physical risk	0.287	0.128	0.026		
Social risk	0.390	0.345	0.152	0.215	

**Table 7:** Results for structural equation modelling.

	Direct Effect	Indirect Effect	Total Effect	Cohen’s f^2^	T Statistics	*p* Values	Supported?
Physical risk → Consumption frequency	β = 0.064		0.064	0.004	1.813	*p* = 0.070	no
Social risk → Consumption frequency	β = −0.026		−0.026	0.001	0.732	*p* = 0.465	no
Psychological risk → Consumption frequency	β = −0.326		−0.326	0.102	9.868	*p* < 0.001	yes
Functional risk → Consumption frequency	β = −0.081	β = −0.117	−0.197	0.006	2.026	*p* = 0.043	yes
Functional risk → Psychological risk	β = 0.327		0.327	0.120	8.129	*p* < 0.001	yes
Functional risk → Social risk	β = 0.389		0.389	0.179	10,014	*p* < 0.001	yes

Model fit: SRMR = 0.061, NFI = 0.856.

**Table 8:** Coefficient determination (R^2^), Adjusted R^2^ and Q^2^

Construct	R^2^	Adjusted R^2^	Q^2^
Physical risk			
Social risk	0.152	0.151	0.064
Psychological risk	0.107	0.106	0.043
Functional risk			
Consumption frequency	0.133	0.130	0.102
